# Clay-reinforced PVC composites and nanocomposites

**DOI:** 10.1016/j.heliyon.2024.e29196

**Published:** 2024-04-06

**Authors:** Seyyed Behnam Abdollahi Boraei, Behnaz Bakhshandeh, Fatemeh Mohammadzadeh, Dorrin Mohtadi Haghighi, Zahra Mohammadpour

**Affiliations:** a Department of Biotechnology, College of Science, University of Tehran, Tehran, Iran; b Biomaterials and Tissue Engineering Department, Breast Cancer Research Center, Motamed Cancer Institute, ACECR, Tehran, 1517964311, Iran; c Department of Polymer Engineering and Color Technology, Amirkabir University of Technology (Tehran Polytechnic), Tehran, Iran; d Department of Pharmaceutics, Faculty of Pharmacy, Tehran University of Medical Sciences, Tehran, Iran

**Keywords:** nanoclay, Composite, aluminosilicates, packaging, thermoplastic

## Abstract

Clay-reinforced polyvinyl chloride (PVC) composites and nanocomposites are one of the newest and most important compounds studied and used in various applications, including the biomedical, automotive industry, water treatment, packaging, fire retarding, and construction. The most important clays used in the synthesis of these composites are Bentonite, Montmorillonite, Kaolinite, and Illite. The addition of these nanoclays to the PVC matrix improves mechanical properties, thermal stability, and yellowness index properties. In this chapter, a detailed study of PVC and its properties, types of nanoclays and their properties, modification of nanoclays, production methods of composites, and nanocomposites of PVC/clay, their characterization, and applications have been performed. Herein, the types, properties, and applications of PVC/clay nanocomposites, as well as their challenges and future remarks, are reviewed.

## Graphical abstract

**Figure:**
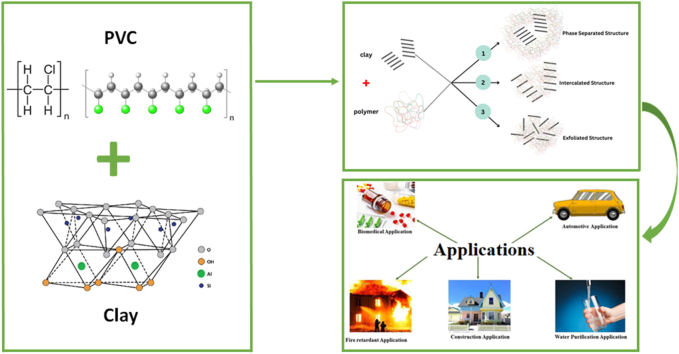

## Abbreviations

Carbon nanotubes: CNTs
Char residue: CR
Chlorhexidine acetate: CHA
Dioctyl Phthalate: DOP
Glass transition temperature: Tg
Halloysite nanotubes: HNTs
Heat release rate: HRR
High-resolution TEM: HRTEM
Layered double hydroxides: LDHs
Limiting Oxygen Index: LOI
Melt Compounding: MC
Montmorillonite: MMT
Organically modified MMT: OMMT
Organo-intercalated LDH: I-LDH
Peak heat release rate: PHRR
Poly(ε-caprolactone): PCL
Polyvinyl acetate: PVA
Polyvinyl chloride: PVC
Scanning electron microscopy: SEM
Silver nanoparticles: Ag NPs
Sodium dodecanoate: SDD
Sodium lauryl sulfate: SDS
Sodium monododecyl phosphate: SMDP
Solution blending: SB
Time to ignition: TTI
Total heat release: THR
Total smoke production: TSP
Transmission electron microscopy: TEM
Vinyl chloride monomer: VCM
X-ray diffraction: XRD

## 1 Introduction

Polyvinyl chloride (PVC) is a prominent thermoplastic material known for its affordability and a wide range of characteristics, including outstanding insulating capabilities, resistance to chemicals, ease of use, and fire resistance [1,2]. It is utilized in construction [3], electronics [4], automotive [5,6], packaging [7,8], optoelectronic [[9], [10], [11], [12]], and healthcare [13]. Although PVC is widely applicable, it nevertheless encounters obstacles, such as poor thermal stability and brittleness, which can result in discoloration and the creation of conjugated polyene sequences [14]. These limitations restrict its possible applications. Nevertheless, continuous research endeavors strive to overcome these limitations by creating pioneering PVC products with enhanced characteristics, consequently broadening their use and augmenting their worth in diverse sectors. To overcome these limitations, one can utilize different nanofillers, including clays, calcium carbonate, silica, talc, layered double hydroxides (LDHs), carbon nanotubes (CNT), mica, vermiculite, and so on, in the production of PVC polymer nanocomposites [15]. This approach offers several benefits, such as enhanced mechanical strength, hardness, heat resistance, barrier properties, etc. [15].

Clay minerals are layered aluminosilicates showing promise as sorbents in environmental remediation [16], food packaging [17], biomedicine [[18], [19], [20], [21], [22]], and construction [23] among others [24,25]. Such widened utility stems from their accessibility, cost-effectiveness, high specific surface areas, cation-exchange ability, and multilayered organization. Nanoclays have shown exceptional effectiveness as reinforcing agents in polymer-based nanocomposites, with minor incorporation of clay enhancing the physicochemical properties of the polymer structure [26,27]. The manufacturing of clay-based polymer nanocomposites can result in the development of intercalated or exfoliated structures [28], which enhance characteristics such as thermal stability, mechanical strength, and barrier efficiency [29]. In the existing literature, several review articles have explored polymer-clay nanocomposites focusing on specific aspects [30,31]. For instance, some reviews delve into specialized applications such as water treatment [[32], [33], [34], [35], [36], [37], [38]], biomedical uses [39], automotive and construction industries [40,41], and lithium-ion batteries [42]. Others narrow their scope to individual clay types, including montmorillonite (MMT) [43,44], halloysite [[45], [46], [47]], and sepiolite [48].

In the present review article, we have focused on the nanocomposites of PVC and various clay types. The potential of developing customized PVC nanocomposites with enhanced characteristics is provided with a particular focus on recent research conducted within the past 5–6 years. We discussed essential characteristics of PVC-clay nanocomposites, including morphology, thermal stability, mechanical properties, fire retarding, and the yellowness index. Our review extends beyond characterization to explore practical applications. Despite the broader potential of PVC-clay nanocomposites in electromagnetic interference shielding [49], cable sheath application [50], oil sorption [51], shock and vibration damping applications [52], and heterogeneous bipolar membranes [53], our focus within this review is automotive industry, packaging, biomedicine, water purification, and building/construction, aiming to offer a broader perspective on the fascinating world of polymer clay nanocomposites.

## 2 PVC characterization

PVC is a thermoplastic polymer that can be combined with various additives to improve its properties. The most important characteristics are morphology, thermal stability, mechanical properties, and toxicity, as discussed below.

### 2.1 Morphology

In suspension polymerization of PVC, the polymer is added to an aqueous phase consisting of demineralized water, buffer salts, and a stabilizer [54]. After adding an oil-soluble initiator, thermal degradation leads to the formation of short-length PVC chains in the vinyl chloride monomer (VCM) phase. Alteration in PVC morphology is associated with the reactor's pressure during conversion. In less than 0.1% of conversion, the formation of nano-domains can be observed. Conversion values of 1–5% result in the coagulation of nano-domains and the formation of primary particles. In the conversion of 5–30%, the growth of primary particles by coagulation and polymerization occurs until the formation of a three-dimensional network. In 50–70% conversion, the number and size of primary particles are increased until the filling of the total volume with polymer particles. In the final stage, particles are packed simultaneously and cannot move freely. In this process, a single droplet named sub-grain is encompassed by a membrane during polymerization. The thickness of this porous skin is about 10 nm. A decrease in the pore size of this skin increases conversion. Folding of the skin and shrinkage of sub-grains cause a decrease in reactor pressure [55].

Size, shape, and porosity (morphology of polymer) depend on the stiffness and folding of the skin, which is influenced by the suspending agent itself [56]. When skin is toughening enough, final particles consist of one sub-grain, and this toughness facilitates plasticizer uptake, hinders the collapse of particles, and maintains the porosity of the structure. However, when the skin is not tough enough, it cannot prevent coalescence, and thus multicellular particles are produced [56,57].

Morphology is affected by grain porosity, grain shape (spherical, irregular), pore size distribution, grain size distribution, and grain internal pore accessibility, which are influenced by agitation rate, polymerization temperature, concentration and type of suspending agent, viscosity, and initiator [57].

Coalescence becomes a dominant phenomenon as the agitation rate is decreased. Therefore, large multicellular grains are obtained. If the agitation speed is increased, particle size is reduced. An increase in agitation rate higher than a particular limit results in a significant reduction in particle size, which suffers from insufficient stabilization that propels the coalescence of particles and the production of multicellular, extended, irregular shapes and highly porous structures [58].

Increasing the volume of the dispersant phase leads to an increase in the number of droplets. Therefore, the absorption of a suspending agent by each droplet lessens, resulting in the formation of larger particles consisting of fused primary particles with low porosity [59].

Elevated droplet coalescence rate and formation of contracted particles with low porosity will be achieved by increasing the temperature [60].

In suspension polymerization, two main suspending agents, including water-soluble polymers (cellulose derivatives) and partially hydrolyzed polyvinyl acetate (PVA), are employed. By increasing the concentration of PVA, particles with a narrower size distribution will be acquired until they reach a particular limit. After that point, the increase in PVA concentration does not affect PVC particle size. Utilizing PVA with a high degree of hydrolysis causes a reduction in PVA absorption to the water-VC interface, an increase of interfacial tension, coalescence of droplets, and skin toughening that results in shrinkage and fusion of particles and formation of a spherical, irregular, and low porous structure. On the other hand, irregular particles will be captured by employing PVA with a low degree of hydrolysis. Hydroxy propyl methyl cellulose with low molecular weight and an increase of its partition coefficient between water and VC phase gives highly porous particles [59].

If the concentration of the initiator is increased, the diameter of the particles will be diminished. In the system in which the initiator is pre-dispersed in the aqueous phase, the transition into a monomer droplet is incomplete, and thus its non-uniform distribution results in non-uniform particle size. When the initiator is pre-dispersed in the VCM phase, a uniform polymerization rate will be observed in all droplets. Therefore, particles with the same shape and size are procured [61]. Elevation of concentration of some fillers, such as organically modified MMT (OMMT), lessens the porosity of PVC particles [62].

At low viscosity, the protective effect of the stabilizer is incomplete, and thus high coalescence of droplets gives irregular multicellular particles. At large viscosity, particle size is reduced until it reaches a particular point and then is increased again, leading to the production of large unicellular particles since the formation of an unfavorable gel in an aqueous phase lessens the activity of the stabilizer [59].

### 2.2 Thermal stability

At 100 C°, PVC undergoes degradation. The dehydrochlorination starts as HCl is emitted from the structure that autocatalyzes the reaction, resulting in the formation of polyene structures. The degradation follows with secondary reactions such as cross-linking that lead to aromatic compound formation, solubility reduction, deterioration of mechanical properties, and coloring [63]. The degradation reaction is assumed to be a chain reaction consisting of 3 steps: initiation, propagation, and termination. During initiation, loss of HCl from different segments of the molecule takes place and gives allylic chloride groups. During propagation, allylic chlorides are converted into polyenes. During termination, polyenes undergo intra- or intermolecular cyclization [63].

To analyze HCl evolution, the PVC sample is heated under constant temperature and N_2_ flow. The amount of HCl is determined by conductometry after absorbance in water. The thermogravimetric method is used to measure weight loss. It is believed that, at 200 C°, the structure loses only volatile compounds, such as benzene and HCl. The difference between total weight loss and weight loss due to HCl evolution gives the amount of benzene [64].

PVC impurities do not contribute to the polymer's instability since, after re-precipitation, stability shows no increase. Tertiary chlorines of branch carbons and chlorine adjacent to internal double bonds are responsible for PVC thermal instability. The effect of tertiary chlorines is more significant than other groups due to higher concentrations [65].

### 2.3 Mechanical properties

The most important mechanical properties are elongation at break, modulus, impact strength, and tensile strength. At lower molecular weight values, an increase in molecular weight results in an enhancement of mechanical properties. The mechanical properties of PVC are influenced by its morphology. Other important factors affecting mechanical properties are free volume, crystallinity, and orientation [66]. PVC shows a necking process and large deformation in tension. At low temperatures, brittle fractures appear. At normal temperatures, visible crazes are observed on the surface of the rigid PVC, and then by increasing the stress, necking and extension occur [67]. A study evaluated the effect of various parameters on the mechanical properties of PVC foam [68]. It showed the linearity of the stress-strain curve for PVC foams with different densities. The tensile behavior of PVC with a higher degree of cross-linking is similar to PVC with a lower degree of cross-linking, which indicates the cross-linking level does not affect the stiffness and tensile strength. Although density plays an important role in mechanical properties, PVC foams with higher density have higher tensile strength, stiffness, and modulus. Fracture behavior is influenced by loading rate, cross-linking, specimen size, foam density, cell orientation, solid material, and crack length.

It is demonstrated that the width plays no role in fracture toughness. On the other hand, specimen height is a critical parameter. Fracture toughness is decreased considerably as the height of the specimen is increased until it reaches 25 mm, followed by decreasing at a slower rate until reaching 32.5 mm and remains constant afterward.

Increasing in fracture toughness is observed due to the loading rate increase. It is indicated that 3.5 times increase in foam density causes seven times increase in fracture toughness.

Levels of cross-linking play a critical role in fracture toughness. Lower fracture toughness is obtained with a higher degree of cross-linking. It is evident that when the crack is parallel to the rise direction, the fracture toughness is higher than the flow direction.

It is displaced that fracture toughness is steady up to a particular crack length and then is decreased beyond it. There are two major types of PVC. The mechanical properties of these PVCs are discussed below.

1 Rigid PVC (PVC–U):

The glass transition temperature (T_g_) of PVC-U is 80 C° which explains its rigidity at room temperature. Since T_g_ is above room temperature, impact properties are poor while strength and modulus are high. The chlorine atom is responsible for the high density of rigid PVC [69].

2 Flexible PVC (PVC–P):

T_g_ of PVC-P is below room temperature. Therefore, it is flexible. The type and amount of plasticizer determine its mechanical properties [69].

### 2.4 Toxicity

The toxicity of PVC is mainly associated with decomposition products, including HCl, benzene, and unsaturated hydrocarbons. Carbon dioxide, carbon monoxide, and water produced in the presence of oxygen are the most common combustion products. Carbon monoxide is an asphyxiant gas that can cause death after exposure. HCl could result in sensory and pulmonary irritation. The degradation products of PVC are shown to be more potent when it is compared with pure HCl. Some studies indicated that at higher concentrations, eye damage might be observed. The LC_50_ calculated for PVC decomposition products varies between 17 mg/L in the flaming mode and 20 mg/L in the non-flaming mode. It revealed that the PVC pyrolysis products are less toxic than other building materials. In addition, combustion products of PVC additives can cause toxicity as well. Zinc ferrocyanide as an additive causes toxicity due to the reaction between HCl and ferrocyanide, resulting in the formation of hydrogen cyanide [70].

## 3 Clay

Clay is the most common natural mineral filler, followed by phyllosilicate, metal oxides (alkali, alkaline earth soils, magnesium, etc.), and organic matter [27]. A common feature of all crystalline clay particles is the fine-grained natural structure in sheet geometry [71]. Clay particles differ in mineralogy and particle size [72]. Clay particles have cation exchange characteristics. The cation exchange capacity of clay particles is responsible for thixotropy, swelling, and hydration. Cation exchange capacity is affected by pH, structural order, and contaminants [73]. Researchers have focused on clay particles’ distinctive structure and surface features in recent years, such as surface charge, cation exchange capacity, mechanical and chemical stability, plenty of supply, and cost [73]. Clay is classified into MMT, kaolinite, illite, chlorite, and bentonite (Table 1) [74].

**Table 1:** Features of the main categories of clay.

Table 1. Classification of clay materials				
Group Name	**General Formula**	**Layer Type**	**Cation Exchange Capacity (mEq/100q)**	**References**
Bentonite	Al_2_Si_2_O_5_(OH)_4_	2:1	80-130 (Sodium Bentonite) 40-70 (Calcium Bentonite)	[75]
MMT	(Na,Ca)_0.33_(Al,Mg)_2_(Si_4_O_10_)(OH)_2_·nH_2_O	2:1	80–100	[76]
Kaolinite	Al_2_(OH)_4_Si_2_O_5_	1:1	5	[77]
Illite	(K, H_3_O) (Al,Mg,Fe)_2_(Si,Al)_4_O_10_ (OH)_2_. (H_2_O)	2:1	25–100	[73]
Chlorite	(Mg,Fe)_3_(Si,Al)_4_O_10_(OH)_2_· (Mg,Fe)_3_(OH)_6_	2:1:1	10–40	[73]

**Fig. 1:**
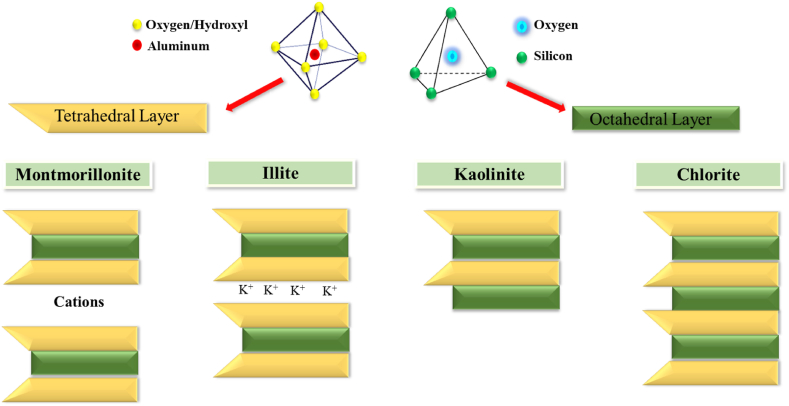
The structure of different types of clays (four types of clays described) is created by the different placement of tetrahedral (trapezoidal) and octahedral (square) layers on top of each other.

They differ in particle size, chemical content, layer structure, and ion replacement. Clay particles have a sheet structure consisting of a tetrahedral layer of oxide silicate and an octahedral layer of aluminum hydroxide. Clays are classified as 1: 1, 2: 1, and 2: 1: 1. Typical interlayer spacing is 0.7 nm for Type 1 clays. The interlayer space is electrically neutral because it lacks cations. The sheet's thickness and length are 10 nm and several hundred nanometers, respectively. Kaolinite is a 1:1 clay, containing two tetrahedral layers with an octahedral layer between them. The compacted layers are 1 nm apart. Isomorphic substitution occurs when a mineral particle crystal component replaces another without changing the chemical structure. Isomorphic substitution creates a surface charge that is balanced by exchangeable cations. In water, cation exchange in the interlayer gap induces swelling. This category includes MMT and bentonite. The 2: 1: 1 clay sheets are an octahedral layer close to the 2: 1 construction. The interlayer space is either empty or hydrated. Illite and chlorite are 2: 1 sheets [71,73,77]. Clay is also classified as two-sided or three-sided. In two-sided clays, all octahedral cavities are divalent (Fe^2+^ and Mg^2+^), but in three-sided clays, two-thirds are trivalent (Al^3+^ and Fe^3+^) [78]. The structure of different types of clay is shown in Fig. 1.

### 3.1 Bentonite

Bentonite is a highly colloidal, negatively charged polymeric clay found almost anywhere. It belongs to the smectite family and is highly colloidal and negatively charged [79]. Bentonite is formed due to the hydrothermal and diagenetic modification of obsidian and the formation of sediments in saline environments. Bentonite is primarily composed of MMT, but it may also contain quartz, feldspar, calcite, illite, mica, and cristobalite, depending on its composition [80]. Because of the presence of MMT in its composition, this particle can expand. However, it should be noted that the presence of impurities reduces thermal stability, which is why bentonite particles must be filtered before use [81]. The number of components and cations that can be transferred between the interlayer space and the bentonite's surface influences its physical properties. In the presence of alumina, cation exchange occurs primarily in the octahedral layer of alumina in bentonites and to a lesser extent in the silicate layer. This material has a small particle size, mesoporous structure, abundance in nature, low price, and availability, as well as desired properties such as high cation exchange capacity, swelling, thixotropic gel formation, good thermal stability, low hydraulic conductivity, high shear and compressive strength, and high bond strength [71,79]. Because of its adsorbent properties and flexibility in the dry state, bentonite is widely used in manufacturing health products, food processing, paint expanding agents, and ceramic additives [79].

Bentonite is classified into three groups based on its exchangeable sodium and calcium cation ratio, swelling index, and pH: The three types of bentonites are natural sodium bentonite, natural calcium bentonite, and active calcium bentonite (sodium-calcium). Natural sodium bentonite is relatively rare and has moderate and similar ratios of exchangeable sodium and calcium and about 10–20% of various minerals such as feldspar, gypsum, silica, and calcite. Natural sodium bentonite has moderate and similar exchangeable sodium and calcium ratios and 10–20% of minerals such as feldspar, gypsum, silica, and calcite. This category's characteristics include optimal viscosity, high dispersibility in the polymer matrix, pH close to 9, high swelling capacity, and thixotropic properties. It is the most common type of bentonite found in many parts of the world, and it is mainly composed of calcium carbonate. The salt content of this group is exceedingly low, while the exchangeable calcium content is exceptionally high. Calcium bentonites offer several characteristics: high adsorption capacity, bleaching ability, a nearly neutral pH, binding strength, and a low swelling index. Activated calcium bentonite may be made by reacting natural calcium bentonite with sodium carbonate; however, the quantity of exchangeable sodium in activated calcium bentonite depends on the extent of activation. An ion exchange process can convert calcium bentonite to sodium bentonite [78].

### 3.2 MMT

MMT, a member of the smectite family, is a hydrophilic phyllosilicate with a thickness of 1 nm and three layers per sheet, with each sheet consisting of three layers [71]. An octahedral alumina layer is sandwiched between two layers of tetrahedral silica in these particles, with water molecules, hydroxyl ions, and cations filling the space between the layers [82]. When adjacent layers form van der Waals, electrostatic, and hydrogen bonds with one another, a primary particle is formed. Weak electrostatic forces cause brittle particles. The secondary MMT particles, which are micrometer-sized, are formed due to the aggregation of the original particles [83]. The layer's negative charge of particles is balanced by the cations residing between the layers [27].

Isomorphic substitution can occur in MMT, with Si^4+^ replacing Al^3+^ in the Al^3+^ tetrahedral and Fe^2+^, Fe^3+^, or Mg^2+^ replacing Al^3+^ in the Al^3+^ octahedral [84]. MMT is found primarily in China, the United States, Ecuador, Russia, and Pakistan [71]. Its application as a reinforcement agent is due to various features, including high cation exchange capacity, low thickness, high swelling, high surface charge, low cost, and high surface area. With minimal cytotoxicity and high dispersion in MMT hydrophilic polymer matrices, these characteristics have led to its usage as a reinforcement. It has been used to enhance polymer substrates' permeability and thermal and mechanical properties [76,85]. Because of its hydrophilicity, MMT is not suitable for usage in hydrophobic polymers. To address this issue, the MMT level must be modified. MMT's chemical formula can be changed by modifying its structural properties [76].

### 3.3 Kaolinite

Kaolinite, a two-sided inorganic phyllosilicate clay, comprises a four-sided silicate oxide layer and an octahedral layer of aluminum hydroxide, with van der Waals and hydrogen bonds holding the layers together [72]. Structured faults are common in kaolinites. Their occurrence is determined by the environmental conditions that prevailed during their formation, such as temperature, pressure, chemical composition, etc. Impurities in kaolinite include metal oxides such as calcium oxide, potassium oxide, magnesium oxide, sodium oxide, manganese oxide, and components such as quartz and mica, which affect the material's irregularity and particle size [86]. Remember that the higher the impurities concentration, the greater the cation exchange capacity. These reactive functional groups are present inside the octahedral layer of kaolinite and can engage in ion-exchange activities. Because kaolinite has a low cation exchange capacity compared to other 2: 1 clay, it is possible to argue that this clay form has a higher anion exchange capacity than cation exchange capacity [87]. The tightness of the structure and the little isomorphic substitution present can be explained by extreme particle failure and difficulties in separating kaolinite sheets. As a result, the interface between this type of clay and the polymer matrix is poor, and its dispersion in the polymer substrate is inappropriate. The chemical composition of a kaolinite surface, the kind of exchangeable cations present, the charge of the layer underneath it, the nature of the atoms present, and the number, type, and extent of defective sites present all impact its physicochemical features. Kaolinite's excellent physical and chemical properties, as well as its high molecular stability in acid/alkaline environments, surface structure, and non-expansion in a wide range of environments, have led to its use in a wide range of applications, including paint, textile, environment, rubber, cable, and so on [88,89].

### 3.4 Illite

Illite is a kind of clay typically found in sea rocks and is the mineral responsible for the formation of the rock. The most common clay mineral found in marine rocks is illite. Although the structure of illite is exceptionally similar to that of MMT, it varies in that it has an aluminium hydroxide layer following the silicate layer. Illite is formed due to the erosion of feldspars and mica at high pH levels or as a result of the alteration of smectite clays during the geological process's diagenetic stage. There is no alkali in the space between the illite layers, and the amount of aluminium in them is less than the amount of silica between the layers. The presence of illite prevents water from entering the structure and expanding due to interlayer cation interference [71,78].

### 3.5 Chlorite

Chlorite is a mica-like silicate composed of an alumina layer between two silicate layers. A two-dimensional layer of aluminium oxide, iron oxide, or magnesium oxide is bonded by a hydroxyl bond. Chlorite is a mineral that naturally exists in the earth's crust [71,72]. Chlorites have a more significant concentration of hydroxyl groups than illites and kaolinites, which are more reactive. Chlorites are not usually a member of the clay family, and there is no universally applicable formula for determining their composition. It is often porous in structure and has a high permeability to water. The absence of potassium in the interlayer space distinguishes chlorites from smectites and illites in their structural characteristics. It should be emphasized that chlorite does not include any radioactive elements in its structural composition [90].

## 4 Modification of clay particles

One of the challenges in creating clay-based nanocomposites is ensuring adequate particle dispersion in the polymer matrix, which influences the final favourable characteristics. Clay particles agglomerate because of high surface energy and hydrophilicity [73]. Hydrophobic characteristics can be introduced to clay particles by modification, resulting in enhanced dispersion in the polymer matrix [91]. This explanation, of course, does not apply to all clays. Surface modification promotes hydrophilic MMT to disperse in hydrophobic polymers, but modified bentonite does not interact effectively with nonpolar matrices. Bentonite's surface modification promotes its dispersion in polar matrices. Before adding particles to the matrix, the modification procedure must be performed. By modifying the clay particles, the surface's free energy lowers while the wetting capabilities of the polymer matrix rise. To modify the surface of clay particles, researchers employ two physical and chemical approaches [27,73].

### 4.1 Physical modification methods

Physical modification of clay particles refers to the adsorption of modifying agents on clay surfaces without affecting the structure of the clay particles, resulting in a bit of improvement in the physical and chemical properties of the surface. There are very few physical interactions between the clay and the modifiers when following this process. Thermodynamic principles control physical modification. Its advantage is that the clay mineral structure is not altered due to this technique. Still, it has a disadvantage because minimal forces exist between the adsorbed molecules and the clay particles [78,92].

### 4.2 Chemical modification methods

In the chemical modification of clay particles, organic molecules chemically attach to the surface of clay particles, resulting in a strong connection between the clay particles and the matrix [27,93]. Because of the wide range of surface properties obtained by applying a variety of functionalized polymers, polymer-based surface modification is one of the most effective modification technologies known [91,94]. There are two methods for grafting functionalized polymers to the surface. When the functionalized polymers interact with the active groups of a solid substrate, one-step grafting occurs. This method is used to make brushes out of the low-density polymer. In the two-step grafting process, polymerizable monomers or starting molecules are covalently linked to a solid surface, and chains of polymerized monomers or initiator molecules grow from the surface [92].

Another common approach for modification is the employment of cationic and anionic functional groups in an ion exchange process to modify the surface of a particle [95]. Clay exchangeable cations are replaced with organic cations such as quaternary ammonium alkyl ions, phosphonium cations, imidazolium, diazonium salts, and other organic cations in the cation exchange process. The larger the size of the organic cation than the interlayer cations, the greater the distance between the layers. Cationic surfactants can be used in the mineral cation exchange process. These surfactants have an extended hydrophobic aliphatic tail and a positively charged head and cause hydrophobia on mineral surfaces [91,96]. Following the refining process, clay particles with a high aspect ratio and a large surface area have better thermal, permeability, optical, and mechanical properties in the manufactured product [97]. Silylation is another approach for the chemical modification of clay surfaces. In this respect, the hydroxyl groups in the hydrolyzed silane are chemically reacted with the silanol groups on the clay surface and form covalent bonds. Such strong interaction prevents the organic moiety from immigration to the surrounding solutions and provides a durable immobilized organic layer on the clay surface. Other surface modification approaches include acid activation of clay [98,99], salt modification [100], and metal/metal oxide [101,102] modification approaches.

## 5 Manufacture of PVC-clay composite and nanocomposite

Successful preparation of clay/polymer nanocomposites requires the chemical compatibility between the clay materials as the filler and the polymer matrix. Besides, homogenous filler dispersion demands the breakdown of clay on a nanometer scale. Dependent on the synthetic approach, the nanoclay may be intercalated/exfoliated by the polymer or immiscible with the polymer. The favourite state is the uniform dispersion of nanoclay in the polymeric material. The major preparation processes to synthesize clay/polymer composites are categorized into three approaches, namely, melt intercalation, in situ polymerization, and solution blending (SB).

In SB, polymer and clay are dissolved in an appropriate solvent. The solvent diffuses into the interlayer space of clay and expands the distance between the layers (swelling). After mixing the polymer and swollen clay, the polymer replaces the solvent molecules in the galleries of the clay material. Stirring, shear mixing, and ultrasonication are the agitation forces that facilitate clay dispersion in the polymer solution. In the final step, the solvent is removed, leading to the reassembling of the polymer-intercalated clay and the nanocomposite formation [49,95,[103], [104], [105], [106], [107], [108], [109], [110]]. It is noteworthy that the solvent evaporation rate is critical to the quality of the composite films. In a study conducted by Rani et al., PVC/MMT/CuO nanocomposite films of thickness 60–80 μm were synthesized through SB [49]. PVC was dispersed in dimethyl formamide and heated to 60 °C while vigorously stirred. MMT and CuO nanoparticles were dispersed separately in the same solvent by ultrasonication. The solutions of PVC, MMT, and CuO nanoparticles were mixed and stirred for 12h. The dispersion was then poured into a dish and placed in an oven for solvent removal. The thermal properties of the nanocomposite improved compared to PVC. Besides, the film was recommended as an electromagnetic shielding material. The electromagnetic shielding interference efficiency was −30 dB in the X-band and −35 dB in the Ku-band regions. Despite success on the laboratory scale, the SB method is not cost-effective in industrial applications. Besides, the large amounts of toxic organic solvents pose severe environmental effects on a large scale.

Melt blending is another preparation process for clay/PVC nanocomposites [29,95,101,111,112]. In this method, nanoclay fillers and polymer are blended and heated above the polymer softening temperature in an oxygen-free atmosphere. The mixture then undergoes secondary processing, including extrusion, injection molding, compression molding, and blown film molding. The melt blending method is more suitable for industrial purposes. In addition, higher dispersion of polymer/clay is achieved compared to the SB method. Melt blending, however, is suggested to be inappropriate for the processing of organically modified clays, as the organic modifier catalyses dehydrochlorination of PVC at high temperatures. In contrast, clay particles that are modified with anionic surfactants can turn into nanocomposites using the melt blending method. In this respect, Yin et al. synthesized anionic-surfactant-modified lanthanum OMMT (La-OMMT) with superior mechanical properties, smoke reduction, and fire retardancy than MMTs modified by quaternary ammonium salts [113]. The nanocomposite was prepared by mixing PVC, dioctyl phthalate (DOP) (as the plasticizer), titanium dioxide and lead salt (as heat stabilizers), and OMMT in a high-speed stirrer at 120 °C. Subsequently, the samples were compressed to 4 mm and cooled down. In another study, solution and melt blending were combined for the preparation of clay/PVC nanocomposites [114].

In situ polymerization starts with the intercalation of monomers into the layered space of clay, followed by polymerization of the monomer via radiation, heat, or initiators [[115], [116], [117]]. Highly dispersed polymer composites can be prepared in an improved polymerization process, in which clay exfoliation is facilitated by polymer chains growing on the surface of nanoclays. The process is called the surface-initiated controlled/living radical polymerization method, in which a higher control over the polymer's structure, molecular weight, topology, and dispersity is achieved. Furthermore, the side reactions associated with conventional free radical polymerization are reduced, and narrow molecular weight distribution is obtained. Clay/PVC nanocomposites prepared by in situ polymerization are reported [118,119].

## 6 Characterization of PVC-clay composite and nanocomposite

The status of polymer dispersion into the interlayer space of clays, as well as the interactions between two parties, is important in the context of thermal and mechanical stability. X-ray diffraction (XRD), microscopic techniques, and Fourier transform infrared spectroscopy are the main techniques used to evaluate the morphological and structural characteristics of clay/PVC composites and nanocomposites. In this section, some of the important techniques for the nanocomposites’ characterization are discussed. Next, the impact of structural properties on thermal/mechanical characteristics, fire retardancy, and yellowness index are discussed.

### 6.1 Morphology

**Fig. 2:**
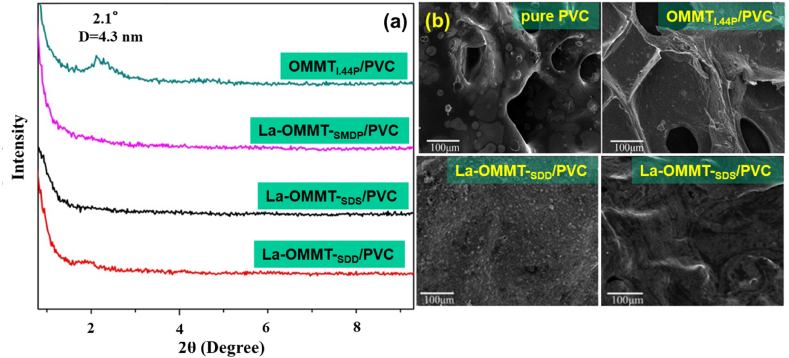
(a) XRD patterns of PVC-based nanocomposites into which organically quaternary-ammonium-modified MMT (OMMT_1.44P_/PVC) and anionic-surfactant-modified lanthanum organic MMT (La-OMMT-_SMDP_/PVC, La-OMMT-_SDS_/PVC, and La-OMMT-_SDD_/PVC) are incorporated; (b) SEM images of the residual degradation products of pure PVC and PVC based nanocomposites after pyrolysis [113].

The morphology of clay/PVC nanocomposites is assessed by various techniques [120]. XRD analysis, wide-angle X-ray scattering, and small-angle X-ray scattering are used to determine if clay platelets are dispersed in the polymer conventionally (microscale dispersion), exfoliated, intercalated, or mixed state (exfoliated and intercalated). In the conventional mode, the polymer and clay are phase-separated such that the microscale clay particles are distributed in the polymer matrix. The intercalated state is the form when polymer chains enter the clay's gallery and increase the interlayer spacing. The exfoliated state is assigned to the complete delamination of the layered structure of clay by polymer chains. The position, shape, and intensity of XRD peaks reveal the structural variation of the nanocomposites. For example, complete delamination of clay is reflected by the disappearance of XRD peaks [121], while intercalation results in the shift of XRD peaks to lower angles and a decrease in peak intensity [49,110,113,122,123]. Fig. 2a displays the XRD pattern of PVC-based nanocomposites prepared by quaternary-ammonium-modified MMT (OMMT_1.44P_/PVC) and anionic-surfactant-modified lanthanum OMMT (La-OMMT-_SMDP_/PVC, La-OMMT-_SDS_/PVC, and La-OMMT-_SDD_/PVC). The peak located at 2θ = 2.1° has shifted toward lower angles with respect to pure Na-MMT, indicating intercalation of the nanoclay by the polymer. In contrast, no XRD peak is observed for the nanocomposites prepared by anionic surfactant, ascribed to complete exfoliation of the nanoclay layers.

The microstructure of the clay/PVC nanocomposites can also be determined by transmission electron microscopy (TEM) or high-resolution TEM (HRTEM). The intercalated or exfoliated state of the layered silicate, the spatial distribution of clay platelets in the polymer matrix, the existence of structural defects or heterogeneities, and atomic arrangement have been studied by TEM/HRTEM. The surface roughness and dispersion degree of clay particles are directly observable by scanning electron microscopy (SEM). Atomic-force microscopy allows for the assessment of surface roughness with higher resolution than SEM, evaluation of particles’ distribution and size, and determining the dispersion status of nanoclays in the polymer matrix. More agglomerates in the TEM image of the nanocomposites, which occur either at high clay contents [121,122] or the incompatibility of the clay material with the PVC matrix [110], reveal poor dispersion of the clay particles in the polymer matrix. The uniform distribution of clay nanolayers in the polymer matrix can be identified by surface roughness deduced from the SEM image. Dutta et al. observed a smooth surface when 5% MMT was present in the polymer nanocomposite [121]. The addition of higher amounts of MMT, however, increased the surface roughness, possibly due to the aggregation of clay particles, which was also verified in TEM micrographs. Fig. 2b shows the morphological changes of PVC in the absence and presence of two clay types (quaternary-ammonium-modified MMT (OMMT_1.44P_/PVC) and anionic-surfactant-modified lanthanum OMMT (La-OMMT_(anionic surfactant)_/PVC) after pyrolysis. The char residue (CR) of pure PVC and OMMT_1.44P_/PVC showed holes in the microstructure, while the surfaces of PVC char modified by anionic surfactants were intact. The continuous layer of the latter inhibited smoke and energy transport, resulting in improved flame retardancy and smoke suppression.

### 6.2 Fire retardancy

PVC, as a widely employed carbon-based material in industrial processes, is combustible, making it a potential hazard in fire accidents. Upon thermal decomposition, it leaves behind a solid carbon residue, with gaseous by-products released. To enhance its fire resistance, plasticizers or additives are incorporated into PVC, resulting in improved fire performance compared to un-plasticized PVC [[124], [125], [126], [127]]. Combining fire retardants with nanoparticles can promote the fire retardancy of the final nanocomposite. Although nanomaterials alone lack self-extinguishing properties, their combination with fire retardants exhibits a synergistic effect that enhances fire retardancy [128]. The incorporation of nanoparticles not only reduces the required amount of fire retardants for effectiveness but also preserves the mechanical properties of the polymer matrix [129]. Nanoparticles effectively mitigate the vulnerability of polymers to flame and high temperatures, serving as nanofillers for fire retardancy [130].

**Fig. 3:**
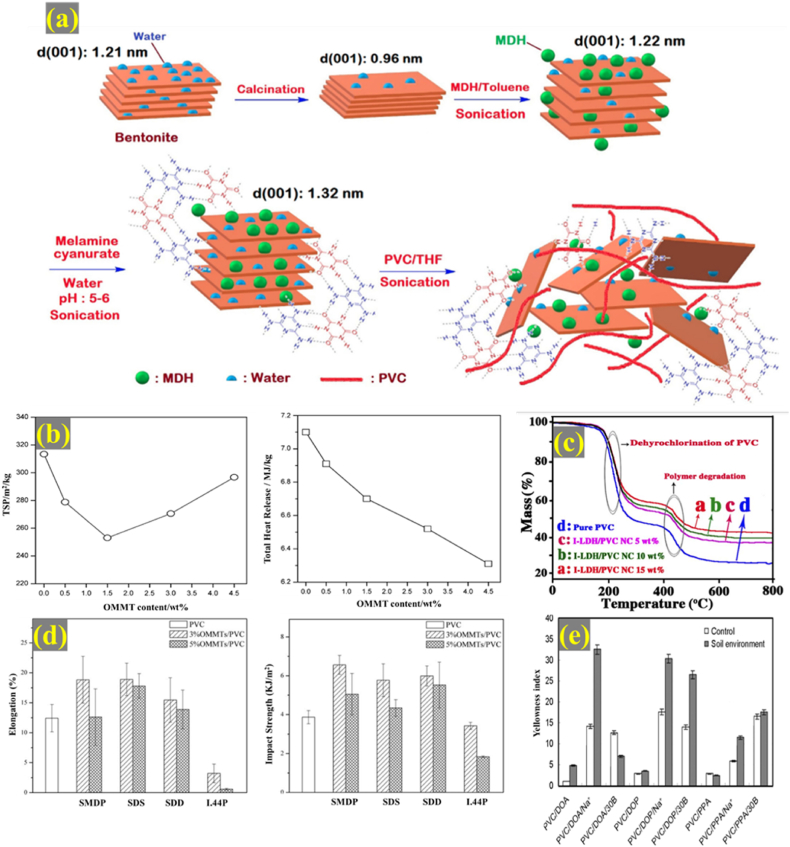
(a) A diagram illustrating the process of preparing a PVC-clay nanocomposite with the addition of both organic (melamine cyanurate (MC)) and inorganic (magnesium dihydroxide (MDH)) fire retardants [132], (b) The effect of clay content on the fire retardancy (TSP and THR) of OMMT/wood flour/PVC nanocomposites [133], (c) Thermogravimetric analysis of organo-intercalated LDH (I-LDH)/PVC nanocomposites comprising 5, 10, and 15% I-LDH [134]; (d) impact strength and elongation of pure PVC, lanthanum OMMT modified by three anionic surfactants (La-OMMT-_SMDP_/PVC, La-OMMT-_SDD_/PVC La-OMMT-_SDS_/PVC), and OMMT modified by dimethyl dioctadecyl ammonium chloride (OMMT-_I.44P_/PVC) [113]; (e) yellowness index of the nanocomposites treated after the soil burial test (different plasticizers DOP, poly(propylene adipate) (PPA), and dioctyl adipate (DOA)) and clays (Cloisite Na+ and Cloisite 30B) were used for the synthesis of clay/PVC nanocomposites) [152].

In this respect, nanoclays have found great interest in improving the fire properties of polymer materials [47,131]. Flammability is measured by cone calorimetry [130]. The measurement of time to ignition (TTI), peak heat release rate (PHRR), heat release rate (HRR), total heat release (THR), CR, total smoke production (TSP), and smoke production rate provides information regarding the fire retardancy of the clay/PVC nanocomposites. In a study led by Hajibeygi et al., a nanohybrid incorporating magnesium dihydroxide and bentonite as flame retardants was prepared (Fig. 3a) [132]. The nanohybrid underwent surface modification with melamine cyanurate, an organic flame retardant, via hydrogen bonding. This addition not only reduced the need for inorganic flame retardant in the polymer matrix but also improved compatibility by evenly dispersing polar layered nanoparticles in non-polar polymers. Incorporating both organic and inorganic fire retardants into PVC decreased PHRR, forming a protective layer on the polymer surface. Adding 8% of the modified nanohybrid to PVC reduced THR by 24.5%, indicating enhanced fire retardancy. Limiting Oxygen Index (LOI) is another parameter for fire retardancy, calculated based on the Van Krevelen equation using the CR value. LOI values demonstrated higher flame resistance with modified bentonite compared to bentonite alone. The water released during MgO formation further increased flame retardancy.

Zhao et al. showed that the inclusion of OMMT into a composite of wood flour and PVC boosts fire resistance and suppresses the smoke emission of the composite (Fig. 3b) [133]. It was observed that the CR value increased, TTI and time to PHRR postponed, and the average HRR and TSP (below 1.5% of OMMT) reduced. The authors proposed that the inorganic shield stemming from the nanoclay acts as a barrier of heat and oxygen and remarkably hinders the combustion of the nanocomposite. Higher amounts of OMMT turned into a thicker shield; therefore, TTI and time to PHRR were delayed. The TSP, however, increased at high quantities of OMMT, possibly due to aggregation of OMMT particles. Besides, by increasing the OMMT content, the HBr gas from the organic modifier (decomCetylalkyl trimethyl amine bromide) accelerated the PVC degradation and raised TSP (Fig. 3b). The value of LOI for active fire retardants is higher than 28. Mallakpour et al. prepared an organo-intercalated LDH/PVC composite using an anionic organic diacid as the intercalant [134]. The value of LOI for different amounts of LDH in the nanocomposite was higher than the pure PVC, revealing the self-extinguishing property of the synthesized materials.

Organic modification of clay particles by quaternary ammonium salts provides compatibility of MMT with PVC. However, the process does not always improve fire retardancy due to its adverse effect on PVC degradation. Alternatively, Yin et al. showed that modification of MMT with anionic surfactant provides compatibility with PVC and prevents its dehydrochlorination [113]. They prepared four clay/PVC nanocomposites; in one, MMT was modified by a cationic surfactant (OMMT-_I.44P_/PVC). In the other three, MMT was modified by anionic surfactants. Among three anionic surfactants, including sodium lauryl sulfate (SDS), sodium dodecanoate (SDD), and sodium monododecyl phosphate (SMDP). Lanthanum was selected as the counterion for the insertion of anionic surfactants into the interlayer space of MMT (La-OMMT-_SDS_/PVC, La-OMMT-_SMDP_/PVC, and La-OMMT-_SDD_/PVC). Unlike OMMT-_I.44P_/PVC, the average HRR, PHRR, and average specific extinction area of the La-OMMT/PVC nanocomposites were lower than that of unmodified PVC. The degradation products of all nanocomposites were collected and analyzed by gas chromatography-mass spectrometry. The authors concluded that La-OMMT/PVC nanocomposites inhibited the conversion of polyenes into cyclic compounds, resulting in smoke reduction and fire retardancy. A list of PVC-clay nanocomposites and their fire resistance properties is presented in Table 2.

**Table 2:** Fire resistance property of PVC-clay nanocomposites.

Clay used	LOI (%)	TTI (s)	HRR (kW/m^2^)	THR (MJ/m^2^)	PHRR (kW/m^2^)	CR	TSP (m^2^/s)	Mass loss %	Ref
La-OMMT-SMDP			62.4 (70.3)		131.5 (156.5)				[113]
Melamine cyanurate modified Mg(OH)_2_@Bentonite	33.4 (28.1)^a^			11.4 ± 0.2 (14.2 ± 0.3)	90.5 ± 1.4 (144.1 ± 2.3)				[132]
Rise husk ash/MMT/epoxidized soybean oil	66.6								[135]
Rice husk/MMT (5phr)	50.0								[121]
Microcrystalline cellulose/rise husk/MMT (3 phr)	50.0								[136]
Halloysite nanotube (HNT)	46.6 (23.5)	62.7 (33.4)	377.5 (440.6)	250.7 (305.9)	536.6 (612.3)				[137]
MMT-Fe_2_O_3_					61.5 (141.8)		3740 (12200)		[101]
Nanomer® I.44P		71 ± 5 (60 ± 20)		120 ± 6 (90 ± 5)	138 ± 3 (282 ± 10)			76 ± 0 (87 ± 3)	[138]

a Values within parentheses represent the experimental quantity in the absence of clay material.

### 6.3 Thermal stability

Thermal properties are important aspects of PVC-based products in practical applications. In a process called chain-stripping, PVC degradation, in its first step, leads to the evolution of HCl and the formation of alternating double bonds along the polymer backbone. In the second step, the double bonds undergo cyclization reactions and smoke formation. According to Awad et al., the addition of nanoclays interferes with the cyclization process, which contributes to smoke reduction [139]. The mass loss upon thermal heating determines the thermal stability of polymer nanocomposites. Initial decomposition temperature, maximum decomposition temperature, decomposition temperature at different weight loss percentages, and residual weight are the experimental parameters quantified to evaluate thermal stability. To this end, differential thermal analysis, thermogravimetric analysis, and differential scanning calorimetry are techniques used to assess thermal stability.

The nature of the intercalant is one factor that determines the thermal stability of clay/PVC nanocomposites. For example, Madaleno et al. showed that the types of clays’ interlayer cations and nanocomposite preparation determine the thermal properties. They employed SB and SB + melt compounding (SB + MC) methods to synthesize Na-MMT and OMMT [114]. In general, the onset of thermal decomposition of nanocomposites prepared by the SB + MC method was higher than those synthesized by SB, as high processing temperature, in the former approach, facilitates the dispersion of clay particles. The onset thermal decomposition of OMMT/PVC was lower than Na-MMT and PVC because OMMT accelerates the PVC degradation. However, the mass loss of the nanocomposites upon thermal treatment was less than that of the PVC. Therefore, clay particles as barriers induce a thermal isolation effect and delay the diffusion of volatile components, which increases thermal stability. Other studies have also verified that modification of nanoclays with quaternary ammonium salts as the organic modifiers accelerates the dehydrochlorination of PVC and deteriorates the thermal stability [113,139,140]. Improved thermal stability of clay/PVC nanocomposites than the unmodified polymer has been reported (Fig. 3c) [113,121,134,141,142]. It has been proposed that the char, which originated from the thermal treatment of the clay part of the nanocomposite, obstructs volatile compounds from leaving the nanocomposite.

Another parameter that affects the thermal stability of clay/PVC nanocomposite is the extent of the clay dispersion in the polymer matrix. A higher degree of clay exfoliation results in improved thermal stability. In a report by Turhan et al., kaolinite/PVC nanocomposites were prepared by the SB method [143]. DMSO was used to modify kaolinite such that the clay's interlayer space increased and particles' dispersion in the polymer improved. They observed that nanoscale compounding of the clay particles with PVC (natural (kaolinite/PVC) and modified kaolinite (K_DMSO_/PVC)) enhanced thermal stability. Specifically, the mass loss temperature of the K_DMSO_/PVC shifted to higher values than kaolinite/PVC. The reason was ascribed to the larger surface area of K_DMSO_/PVC than that of kaolinite/PVC, resulting in a stronger interaction between the components in the former nanocomposite. Besides, the thermal stability of the nanocomposites was superior to pure PVC. It was suggested that HCl evolve from dehydrochlorination of PVC to autocatalyze the thermal degradation of PVC. The hydroxyl groups of kaolinite inside the nanocomposites react with HCl and hinder the degradation process. In addition, a higher amount of char in the presence of kaolinite prevents the diffusion of volatile decomposition products from getting out of the composite. Contrarily, Dutta et al. showed that adding a high amount of clay to the polymer composite lowers the onset of thermal decomposition and maximum decomposition temperature, compared to unmodified composite, due to poor dispersion of the clay particles in the composite [121].

### 6.4 Mechanical properties

The mechanical properties of clay/PVC nanocomposites are measured by experimental parameters, including tensile strength, Young's modulus, and impact strength, among others. The addition of clay to the matrix of PVC, in most cases, reinforces the mechanical properties of the resulting nanocomposite [121,136,144]. The observed effect is attributed to the interfacial interaction between the exfoliated nanoclay and the PVC matrix. Adding more clay beyond a critical amount weakens the mechanical properties due to clay particles' agglomeration, resulting in decreased interfacial interaction between the clay and polymer matrix and decreased effective stress transfer within the polymer nanocomposite. An improvement in the mechanical performance of clay/PVC nanocomposites makes them suitable alternatives to commercial materials used for industrial applications. For example, Dan-asabe et al. showed that, compared to commercial carbon steel and PVC, the Kankara clay/PVC composite had better mechanical performance and lower price/weight in piping applications [145]. Highly exfoliated clay structures that establish a strong interaction with the polymer matrix exhibit superior mechanical properties to those in which the clay particles aggregate [110]. A study showed that the addition of LDHs and HNTs to the PVC polymer contributes to the improvement of mechanical stability [122]. Larger interlayer spacing of LDH/PVC composite than HNT/PVC results in higher dispersion of the former in the polymer matrix and superior mechanical properties.

The interlayer ions of the nanoclays, organic surface modification of clay particles, and the nanocomposites' preparation method determine the mechanical property of the clay/PVC nanocomposites [[146], [147], [148]]. Petersen et al. observed that modification of MMT with tributyl citrate followed by incorporation into diisononyl phthalate-plasticized PVC resulted in inferior mechanical properties to the unmodified composites [146]. They found that extraction of the organic modifier from the surface of clay and dispersion within the plasticized PVC causes the agglomeration of clay particles and decreased performance. Besides, it has been proposed that organic modification of the clays using quaternary ammonium salts accelerates PVC degradation and undermines the mechanical properties [140]. To solve the challenge, Yin et al. suggested that modification of MMT with anionic surfactants increases the compatibility with the polymer matrix and inhibits the dehydrochlorination process, which consequently improves the mechanical stability [113]. Among three anionic surfactants, SDS, SDD, and SMDP, the tensile strength, impact strength, modulus, and elongation of the La-OMMT_SMDP_/PVC was the highest, while the mechanical characteristics of the composite modified by quaternary ammonium salt were inferior to PVC (Fig. 3d). Despite such reports, the effect of clays’ surface modification by quaternary ammonium ions on the mechanical properties of PVC-clay nanocomposites is not conclusive. Kumari et al. [149] reported the opposite observation of Yin et al. in the previous example [113].

Madaleno et al. incorporated Na-MMT and OMMT within the PVC matrix using two methods, SB and SB + MC [114]. While the addition of Na-MMT to PVC by the SB method increased the tensile strength, enhanced OMMT content of the nanocomposite by the same method led to tensile strength reduction. The authors proposed that OMMT particles that are poorly dispersed or agglomerate in the nanocomposite matrix create point defects, the result of which is a reduction in tensile strength. In addition, regardless of the clay type, the nanocomposites that were prepared by SB exhibited a decrease in elongation value. The reason was suggested to be the mobility restraint of the polymer chain imposed by the exfoliated clay particles. Contrarily, the elongation value of OMMT/PVC that was prepared by the SB + MC method increased by 29% with 2 parts per hundred resin of OMMT compared to unfilled PVC. Overall, the study concluded that OMMT/PVC nanocomposites prepared by the SB + MC method and low amounts of OMMT showed a combined upgrading in the mechanical properties, including tensile strength, Young's modulus, and elongation. The increased reinforcement efficiency was ascribed to the shear stress achieved at high temperatures due to the high dispersion of the clay nanoparticles in the PVC matrix.

Another parameter that defines the mechanical properties of polymer nanocomposites is T_g_, which is commonly determined by differential scanning calorimetry. T_g_ depends on the mobility of polymer chains. Dispersion of nanoclay layers inside PVC confines the mobility of polymer chains and increases the final rigidity, leading to an increase in T_g_. According to Izadpanah et al., adding 1% cloisite30B clay to a PVC-based copolymer increased the T_g_ value of the polymer nanocomposite by 16.9 °C [110]. At higher clay contents, no T_g_ was observed, confirming the highly cross-linked network of the nanocomposite. The incorporation of OMMT in the PVC matrix, however, is reported to decrease the T_g_ value [144]. The effective surface area and the degree of clay agglomeration affect the T_g_ value in nanocomposites. A list of parameters used to determine the mechanical properties of PVC-clay nanocomposites is presented in Table 3.

**Table 3:** Mechanical properties of PVC-clay nanocomposites.

Clay used	Flexural properties		Hardness	Young's Modulus	Tensile strength (MPa)	Elongation at break (%)	Tg	Impact strength (kJ/m^2^)	Reference
	Strength (MPa)	Modulus (MPa)							
MMT (5 phr)	16.6 ± 0.25 (13.45 ± 0.4)	1405 ± 8.1 (1264 ± 6.5)	72 ± 2.6 (64.0 ± 4.3)						[121]
MMT/microcrystalline cellulose	11.33 ± 0.09 (7.1 ± 0.09)	38.16 ± 1.18 (37.31 ± 0.82)	70 ± 5.5 (57 ± 4.3)						[136]
Kankara clay	84		61.1	2 GPa	50				[145]
C–30B				416 ± 7 (342 ± 6) MPa	11.05 ± 0.5 (9.85 ± 0.5)	2.92 ± 0.2 (13.5 ± 1)	70.9 (60.7)		[110]
mLDH	23.05				32.09		70.67		[122]
Anionic-surfactant-modified lanthanum OMMT					51.0 (49.0)	18.83 (12.42)		6.6 (3.9)	[113]
Organo-bentonite			70 (55)	1.191 (0.881) MPa	2.91 (1.58)				[149]
OMMT				2467 ± 14 (1951 ± 16) MPa	36 ± 2 (22 ± 1)^a^ MPa	574 ± 10 (504 ± 12)			[150]
Halloysite				1800 ± 16.1 (1630 ± 15.2) MPa		45 ± 7.9 (22.3 ± 12)	94.0 (94.7)	23.4 ± 3.1 (15.6 ± 1.6)	[112]
Mg(OH)_2_@bentonite				4.83 ± 0.16 (2.67 ± 0.11) GPa	66.06 ± 2.67 (43.31 ± 1.26) MPa	6.75 ± 0.12 (6.20 ± 0.09)			[132]
MMT					8.6 ± 0.2 (8.0 ± 0.2) MPa	14.0 ± 0.5 (12.5 ± 0.5)			[53]
a tensile strength at break									

### 6.5 Yellowness index

PVC degradation upon exposure to heat, oxidative conditions, and UV light is characterized by the dehydrochlorination reaction and formation of polyenes, which shifts the polymer color to yellow. Color coordinates or yellowness index are used to describe the extent of color change. Polymer composites with lower thermal stability become yellower after aging [151]. Awad et al. prepared nanocomposites of PVC using hectorite and bentonite as the clay materials, propylene carbonate as the dispersant, and di-isodecyl phthalate as the plasticizer [139]. Based on the shift in the yellowness index, they found that the presence of dispersant and mixing order of the components directly affects the thermal stability of the nanocomposites. Among various formulations, the one prepared by pre-gel of organically modified hectorite in the plasticizer and dispersant with subsequent addition to PVC led to the lowest shift in the yellowness index compared to pure PVC. In another study, the effect of plasticizer and clay type (MMT and OMMT) on the biodeterioration of nanoclay/PVC composites in the soil environment was evaluated (Fig. 3e) [152]. Opaque composites presented a higher shift in the yellowness index. The authors proposed that the migration of clay particles to the surface of the composite is responsible for the opaque appearance of composites after the soil burial test. It decreases the diffusion of water and microorganisms into the composite, therefore inducing less susceptibility toward biodegradation. Composites containing natural MMT showed a higher shift in the yellowness index than those containing OMMT. In another example, the thermal stability of hydrotalcite against organic stabilizers that are commonly used in PVC films was studied. The yellowness index assessed the degree of polymer film discoloration. Hydrotalcite with halogen scavenging capacity neutralized the detrimental effect of evolved HCl, leading to the increased heat resistance of PVC films [153]. In addition to the above characteristics, electromagnetic interference shielding [49] and fouling resistance [123,141] of the clay/PVC nanocomposite are superior to pure PVC.

## 7 Applications of PVC-clay composite and nanocomposite

Due to the wide range of properties of the PVC and the clays and their composites, a wide range of applications can be considered for their composites and nanocomposites. Among these applications, we have focused on automotive, biomedical, packaging, water purification, and construction. In the following sections, each of these applications will be discussed in detail.

### 7.1 Automotive industry application

In general, polymer/clay composites and nanocomposites in the automotive industry can be summarized as follows: 1) Production of lighter vehicles to reduce fuel consumption and consequently reduce carbon dioxide emissions. 2) Increase the comfort of vehicles. 3) Improving the safety of vehicles. 4) Increase the driveability of the vehicles [154]. Toyota is one of the leaders in this field, which started using polymer/clay nanocomposites in the early 1990s. Initially, it used polyamide 6 and clay nanocomposites, which was the company's first commercial nanocomposite [31]. Over time, other companies began to use and produce polymer/clay nanocomposites in the automotive industry [31].

Currently, PVC/clay composite and nanocomposite are used in various applications, such as the automotive industry. In a relevant study, scientists investigated the effect of adding HNTs as a nanoclay to the PVC-based matrix for automotive tire applications, which showed the high potential of this nanocomposite in the automotive application along with the significant improvement of mechanical, thermal, and swelling resistance properties [155]. Also, the use of these nanocomposites and nanotechnology in the production of tires can greatly reduce the emission of carbon dioxide and have a great contribution to the environment [155]. In another study, different types of clays and organo-clays were added to PVC to improve the basic characteristics of PVC and reduce its disadvantages, which include brittleness, and low thermal/thermomechanical resistance [149]. Thermomechanical properties are also one of the most important essential properties of PVC, which can be improved by adding nanoclays. Recently, Cloisite30B and Cloisite15A nanoclays were added to a cross-linked PVC, and very suitable thermomechanical results were obtained which can be of great interest and achievement in automotive applications [156].

### 7.2 Packaging application

PVC is one of the widely used packaging materials due to its versatility, lightweight, and cost-effectiveness. Although PVC is a nondegradable polymer, the lack of viable options for biodegradable polymers in commercial settings makes PVC a competing one for this purpose. The addition of nanoparticles to polymers can improve the mechanical, barrier properties, and antibacterial properties of the polymeric materials [157]. For instance, silver nanoparticles (Ag NPs) have been shown to reduce the microbial growth of minced beef and extend the average shelf life of the food product, when coated on PVC-PE laminates by inkjet printing [158]. Similar results were observed by Azlin-Hasim et al. [159]. The incorporation of ZnO NPs on PVC film has also preserved the quality of fresh-cut apples, leading to a reduction in the fruit decay rate [160]. The effect was ascribed to decreased accumulation of malondialdehyde and inhibition of enzyme activity. Clay materials are rapidly developing as nanofillers in food packaging applications [161,162]. An improvement in mechanical properties, barrier properties, thermal stability, and ethylene scavenging has been the result of nanoclay addition to polymer matrix for packaging purposes [163]. Na.MMT is one of the nanoclays used in polymer nanocomposites. The hydration of sodium ions in the interlayer of MMT makes it hydrophilic, thus, unfavorable for mixing with organophilic polymers. The issue has been addressed by exchanging the sodium ion with cations such as alkylammonium ions. In this respect, Sudhakar et al. embedded alkyl ammonium ion exchange MMT into PVC and observed a decrease in the T_g_ of the polymer [164]. Furthermore, the tensile strength of the nanocomposite was increased by up to 6% of the clay attributed to the proper dispersion of the clay in the polymer matrix. The presence of clay slightly increased the degradation rate of PVC (0.8%). The authors proposed that the hydration of clay layers breaks the intramolecular bonds in the polymer structure, resulting in faster degradation.

In a study conducted by Hadj-Hamou et al., PVC was combined with poly(ε-caprolactone) (PCL), a biodegradable polymer [150]. While non-biodegradable polymers like PVC contribute to the barrier and mechanical properties, the inclusion of biodegradable elements in these bioblends helps mitigate environmental waste concerns. The molecular level miscibility of PVC and PCL is well-documented [165]. The addition of the nanoclay reduced the water vapor permeability of the nanocomposite indicating the enhanced barrier properties. The layered structure of the clay resulted in increased diffusion path length of the gas, forcing it to travel through a tortuous path. Notably, high barrier properties of packaging materials are favored for moisture-sensitive products that require longer shelf life. The modified clays contributed to the antibacterial activity of the nanocomposite. The authors suggested that through the intercalation of the PCL/PVC bioblend into the interlayer space of organoclay, a fraction of the ammonium surfactant, ionically bonded to the negatively charged surfaces of the clay layers, would be released into the culture medium exhibiting bactericidal power.

### 7.3 Biomedical application

Polymer-clay nanocomposites in different forms of films, meshes, hydrogels, scaffolds, and 3D-printed constructs have found biomedical applications in drug delivery, tissue engineering, and antibacterial applications [166]. While Clay nanoparticles are biocompatible [167] at concentrations higher than most of the nanoparticles [168] and their degradation product are nontoxic [169], PVC is a synthetic nondegradable polymer. Even though PVC is largely used in medical devices (intravenous bags and tubings, blood bags, catheters, disposable gloves, and dialysis bags) it is not a common material used in some aspects of biomedicine such as tissue engineering, due to the lack of biocompatibility and biodegradability. For such applications, biodegradable or natural polymers that mimic the properties of native extracellular matrix are preferred to support the cell growth and tissue regeneration process.

**Fig. 4:**
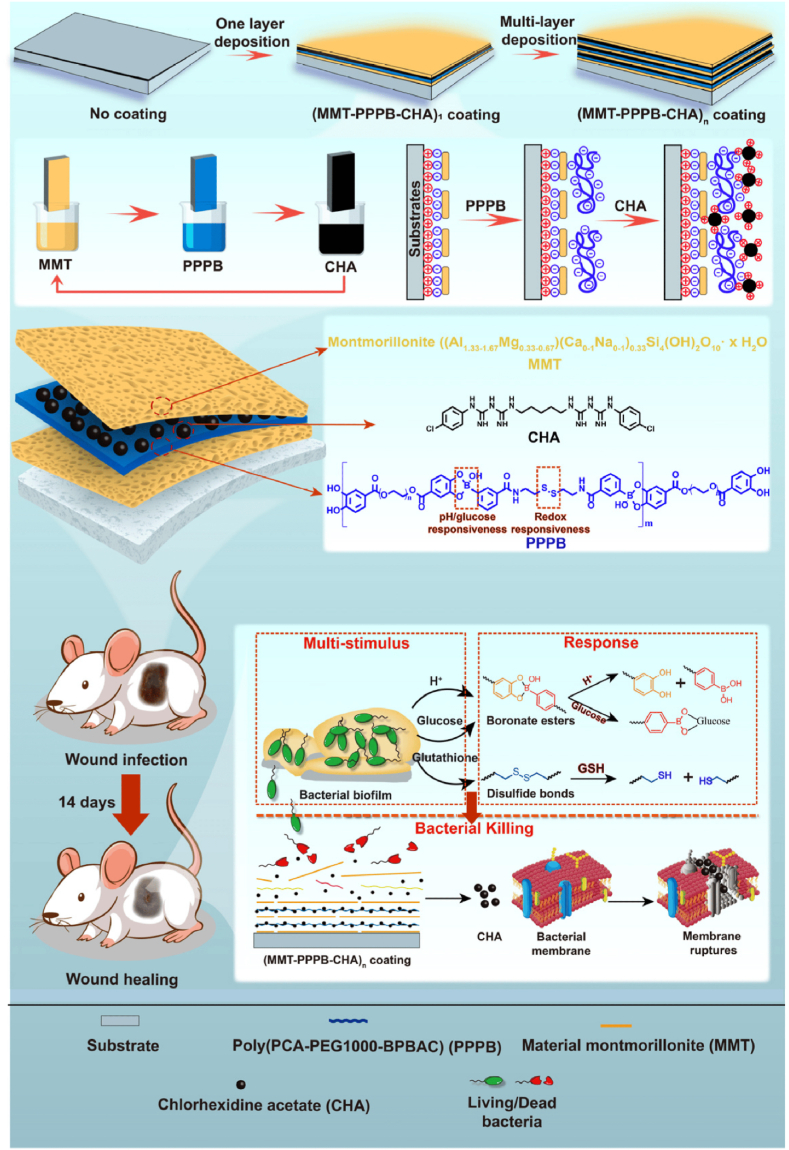
A schematic illustration of multi-responsive antibacterial coating on the surface of PVC. The drug was released in response to acid, glucose, and reducing environment [182].

In light of the extensive use of PVC-based medical devices, researchers have explored the coating of PVC surfaces with various nanoparticles to provide antibacterial coatings. This is especially important because PVC is hydrophobic and favors the attachment and growth of pathogens. Incorporation of Ag NPs-modified chitosan into the polyacrylamide-gelatin network and coating the nanocomposite on the surface of PVC was shown to be promising to prevent biofilm-related infections in the endotracheal tube which is an essential medical device to secure the airway patency in patients undergoing mechanical ventilation or general anesthesia [170]. Composites of PVC with other nanoparticles such as CuO NPs [171], Ag NPs [[172], [173], [174]], ZnO NPs [175,176], modified SiO_2_ nanoparticles [177], silver phosphate NPs [178] gold NPs [179], Cu NPs [180], and SiO_2_–Ag [181] have shown antibacterial activities. In the context of PVC-clay nanocomposites, clay enhances the antibacterial drug loading through electrostatic attraction and intercalation of the drug within its layers. In a study conducted by Li et al., a multilayer antibacterial coating was prepared on the surface of PVC through layer-by-layer self-assembly of MMT, chlorhexidine acetate (CHA), and poly(protocatechuic acid-polyethylene glycol 1000-bis(phenylboronic acid carbamoyl) cystamine) (Fig. 4) [182]. The coating was responsive to acids, glucose or dithiothreitol, enabling the controlled release of CHA and kill more than 99% of *Staphylococcus aureus* and *Escherichia coli* (4 × 10^8^ CFU/mL) within 4 h. In the infected microenvironment, the boronate ester and disulfide bond broke, and the drug was released. In vivo studies revealed that wound healing could be achieved within 14 days. The number of research articles exploring the potential of PVC-clay based nanocomposites for antibacterial activity is rare in the literature [183].

### 7.4 Water purification application

Various methods are utilized for wastewater treatment, including adsorption, precipitation, complexation, membrane filtration, photo- and electro-catalysis, reverse osmosis, and electrodialysis [184]. While these techniques offer benefits, some drawbacks exist, such as high costs, unproven performance at large scales, sludge formation, and the potential for secondary pollution [185]. Adsorption is recognized as a highly efficient and cost-effective technology for wastewater purification. Clay minerals have been employed for this purpose due to their high adsorption capacity, surface area, unique chemical composition with microporosity, layered structure, and nanoscale sizes. However, clays alone may not effectively remove pollutants due to their challenging recovery/regeneration process after use. Additionally, clay particles exhibit a low affinity towards organics because the hydration of their surface hinders the access of organic molecules to their layers [186].

PVC is a cost-effective polymer with suitable chemical properties and physical stability for membrane material production [187]. PVC offers advantages such as film-forming ability, resistance to solvents, bases, acids, solubility in various organic solvents, and longevity. Despite the need for membrane replacement due to fouling issues over time, PVC-based membranes are economically viable for separation processes. The hydrophobic nature of PVC has historically limited its use in pressure-driven membrane processes; however, recent advancements have focused on modifying PVC membranes to enhance hydrophilicity and fouling resistance.

Incorporating nanomaterials into PVC membranes to create nanocomposites shows promise in improving filtration performance by enhancing flux, hydrophilicity, and rejection rates (Table 4). The development of clay-polymer nanocomposites has emerged as a solution to address the limitations of clay minerals and polymeric materials in wastewater treatment [32,33,187]. Primarily used as membranes in water purification applications, PVC-clay nanocomposites have shown potential to overcome previous limitations. For example, in a study conducted by Ahmad et al., a flat-sheet PVC membrane was prepared by blending the polymer with acrylamide-grafted bentonite and employed for oily wastewater separation [106]. 8 wt% of modified bentonite in the membrane structure resulted in an enhanced pure water flux of 293.14 Lm^−2^h^−1^, permeate flux of 123.96 Lm^−2^h^−1^, and oil rejection of around 93.2%. The 4.33-fold increase in the permeate flux of the modified PVC membrane over the plain membrane was attributed to better hydrophilicity, increased pore size, and porosity. Nevertheless, the increased pore size resulted in decreased oil rejection with respect to plain membranes.In another study, Vatanpour et al. developed polyethylenimine (PEI)-functionalized HNTs (HNT-SiO_2_-PEI) and embedded them in the PVC membrane to filter proteins and dyes [188]. They observed that the addition of clay nanoparticles strengthened the membrane by increasing the tensile strength. Besides, an increased amount of clay in the membrane lead to increased flux and rejection rate of BSA and dye. They attributed the observation to increased hydrophilicity of the modified membrane, which was associated with negative surface charges resulting in electrostatic repulsion of the pollution. Other than pressure-driven membrane processes, membranes based on PVC-clay composites have been utilized in water purification based on the pervaporation mechanism [189]. More examples of clay-modified PVC-based nanocomposites that are employed for water purification are presented in Table 4.

**Table 4:** PVC-clay nanocomposites for water purification.

Clay used	Target	Water contact angle (hydrophilicity)	Pure water flux (L.m^−2^h^−1^)	Type of purification	Porosity (%)	Performance	Reference
Acrylamide grafted bentonite	Crude oil	49.1 ± 1.1(80.5 ± 1.9)^a^	293.14 (5.54 times higher than plain PVC membrane)	filtration	71.22 ± 2.55 (40.12 ± 1.02)	Oil rejection >93.2%	[106]
TiO2 decorated functionalized HNTs	methylene blue (MB) and rhodamine B	–	–	Photocatalytic degradation	–	PVC photocatalytic membranes degraded up to 42.37%, 32.76% MB and RB respectively	[190]
HNT-SiO_2_-PEI	BSA and dye	51.7 ± 2.4 (64.6 ± 2.8)	169.1 (37.3)	filtration	72.5 ± 1.8(60.6 ± 1.8)	Rejection of BSA: ≈99 Rejection of Reactive Orange 64: 99.97	[188]
Modified MMTs with folic acid	humic acid	72.0 ± 2 (56 ± 2)	–	filtration	83 ± 1 (70 ± 1)	Rejection of 90%	[123]
CeO_2_ loaded halloysite (HNTs)	humic acid	78.48 ± 3.53(91.3 ± 5.8)	206.42 (127.33)	filtration	52.92 ± 0.73(42.80 ± 3.08)	Rejection of 97.9%	[191]
PF127/bentonite	oil	9.9 (13.9)^b^	1610.0 (1444.1)	filtration	74.05 ± 1.68(72.32 ± 1.67)	Rejection of 98%	[192]
Surface-modified PVC/NanoClay/Tween60	Basic Orange 2	10.0 (73.5)	0.058 (0.036)^c^	filtration	–	Dye removal efficiency was 90%	[193]
Bentonite nanoclay	oil	9.6 ± 2.06 (15.7^)^^b^	607.81	filtration	73.13 ± 8.68 (72.85)	Oil rejection >92.0 %	[108]

a Values in the parantheses are the experimental quantities without clays.

b Dynamic water contact angle at 60 s.

c The unit is mL c^m−2^ s^−1^.

### 7.5 Building and construction

PVC has various applications in the construction industry, which is due to its low cost, high stability, high versatility, and ease of working with it [194]. However, in the construction industry, there is a need for suitable mechanical properties (enough brittleness) of the materials used, as well as high thermal resistance, which is one of the disadvantages of PVC, and these problems must be minimized by novel approaches such as producing PVC composites [195]. In a study, MMT clay was added to PVC with percentages less than 5%, and the results showed the improvement of the desired mechanical properties as well as the stability of PVC [195].

Recently, a novel composite with different ratios of PVC and clay was investigated to strengthen the mechanical properties of concrete, and the results showed an improvement in cement strength, and no decrease in freshness and mechanical properties of cement was observed [196]. In another investigation, Sevinç and Durgun produced a new composite by adding PVC and vermiculite to epoxy resin, which had interesting properties such as reducing thermal conductivity coefficient and reducing water absorption of the produced composite, which are two important parameters in construction applications [197].

## 8 Conclusion and future remarks

PVC, a widely utilized polymer worldwide, has gained attention across numerous industries for its exceptional durability, cost-effectiveness, resistance in harsh environments, and ease of transportation. Despite these advantages, PVC has limitations, including brittleness and restricted thermal stability. An effective approach for addressing these challenges involves utilizing various forms of nanoclay to create PVC/clay nanocomposites. The method of nanocomposite preparation significantly impacts the chemical compatibility between clay and PVC, as well as the even distribution of clay inside the polymer matrix, thereby impacting the final characteristics of the synthesized material. Generally, a minimal loading of nanoclay is sufficient to achieve the excellent development of properties without substantially increasing density, cost, or diminishing light transmission properties.

It's crucial to acknowledge that the technique of clay modification, the clay's surface area, and the clay quantity substantially affect the thermal stability and mechanical properties of the PVC nanocomposites. The inclusion of clay into the PVC matrix, in most cases, enhances fire resistance, reduces smoke emissions, improves thermal stability, boosts flame retardancy, reduces the yellowness index, and imparts self-extinguishing features by creating a mineral barrier against heat and oxygen. However, the impact of clay on the thermal stability of the PVC matrix has been reported to be detrimental in certain cases. Thermal stability and fire-resistance qualities can be influenced by the type of clay modification. While some studies suggest that modification with bulky cations accelerates PVC degradation, others indicate increased thermal stability with the incorporation of anionic surfactants into clay minerals. Therefore, systematic studies are needed to examine the effects of organic modifier chemistry, additives, and production methods on thermal stability and heat transfer mechanisms within the composite structure. The acceleration of PVC degradation and clay aggregation in the PVC matrix can adversely affect mechanical properties. Additionally, the incompatibility between surface modifiers and clay minerals may lead to the migration of the modifier from the clay surface, particle agglomeration, and a compromise in mechanical properties. Furthermore, the chosen process technique impacts the degree of clay dispersion/exfoliation and the resulting mechanical properties.

While PVC stands out as a high-strength thermoplastic with resistance to corrosive liquids, its environmental impact cannot be ignored. Although PVC/clay composites or nanocomposites find practical applications, the non-biodegradability of PVC restricts its use in certain applications, such as tissue engineering. In other circumstances, blending PVC with nanoclays enhances durability, potentially reducing waste generation. In addition, combining nanoparticles with natural fibers and the PVC matrix improves the properties of biodegradable composites, lowers the reliance on thermoplastic, and subsequently reduces waste production and pollution. It has also been observed that clay minerals can accelerate the photodegradation of PVC microplastics. The potential for clay minerals as nanofillers in PVC-based composites may facilitate the degradation of the wasted material in specific chemical conditions, presenting an intriguing area for future research exploration.

## Funding

The author(s) received no financial support for the research, authorship, and/or publication of this article.

## CRediT authorship contribution statement

**Seyyed Behnam Abdollahi Boraei:** Writing – review & editing, Writing – original draft, Visualization, Project administration, Methodology, Investigation.**Behnaz Bakhshandeh:** Writing – review & editing.**Fatemeh Mohammadzadeh:** Writing – review & editing, Visualization, Investigation.**Dorrin Mohtadi Haghighi:** Writing – original draft, Investigation.**Zahra Mohammadpour:** Writing – review & editing, Writing – original draft, Visualization, Investigation, Supervision.

## Declaration of competing interest

The authors declare that they have no known competing financial interests or personal relationships that could have appeared to influence the work reported in this paper.
